# Socioeconomic position interacts with the genetic effect of a *CRP* gene common variant to influence C-reactive protein values

**DOI:** 10.1038/s41598-024-83437-w

**Published:** 2024-12-23

**Authors:** Miriam Cheaib, Nicola Hornung, Nico Dragano, Mirjam Frank, Per Hoffmann, Markus M. Nöthen, Raimund Erbel, Andreas Stang, Börge Schmidt

**Affiliations:** 1 Institute for Medical Informatics, Biometry and Epidemiology, University Hospital Essen, University of Duisburg-Essen, Hufelandstr. 55, 45122 Essen, Germany; 2 Institute of Medical Sociology, Centre for Health and Society, Medical Faculty and University Hospital, University of Düsseldorf, Düsseldorf, Germany; 3 Institute of Human Genetics, University of Bonn, Bonn, Germany; 4 Department of Genomics, Life and Brain Center, University of Bonn, Bonn, Germany; 5 Department of Epidemiology, School of Public Health, Boston University, Boston, USA

**Keywords:** C-reactive protein, *CRP* gene, Interaction, Socioeconomic position, Epidemiology, Epidemiology

## Abstract

Objectives: C-Reactive Protein (CRP) values are partly determined by variation at the *CRP* gene locus, but also influenced by socioeconomic position (SEP) and related lifestyle factors. As gene-by-SEP interactions have been suggested for traits associated with CRP and SEP (e.g., BMI, coronary artery disease), the aim of this study was to investigate the strength of a possible interaction between a *CRP* gene common variant (rs4287174) and SEP in their joint influence on CRP levels in a population-based study sample. Methods: Single nucleotide polymorphism rs4287174 was genotyped in 4065 participants (aged 45–75 years) of the Heinz Nixdorf Recall study, a population-based prospective cohort. SEP indicators (education and income), risk factors (i.e., body mass index (BMI), total cholesterol, diabetes mellitus, coronary artery calcification, current smoking, hypertension, diet, no exercise) and blood serum CRP (mg/dl) were assessed at study baseline. Interaction analysis was based on linear regression and on stratified analyses (genetic effect stratified by SEP and vice versa) adjusted for age and sex using log_e_(CRP + 1) as dependent variable. Results: Low SEP and rs4287174 T allele were both associated with higher CRP values. The strongest genetic effect was observed in the lowest educational group (≤ 10 years of education) with an exp(β) indicating 1.058-fold (95%-CI: 1.018; 1.100) average CRP values per additional T allele, while in the highest educational group (≥ 18 years) the association was considerably less strong (exp(β): 1.005 (95%-CI: 0.975; 1.037)). After including rs4287174-by-education interaction terms in the regression analysis, interaction was indicated suggesting stronger genetic effects on CRP in low SEP groups (exp(β_interaction_): 1.056 (95%-CI: 1.005; 1.108); *p* = 0.029). The observed interaction did not seem to be substantially mediated by the risk factors included in the analysis. No indication for rs4287174-by-income interaction was observed. Conclusion: Results imply that genetic effects of the CRP locus are modified by education as an indicator of life course SEP.

**Supplementary Information:**

The online version contains supplementary material available at 10.1038/s41598-024-83437-w.

## Introduction

C-reacting Protein (CRP) is an acute-phase protein^1^. It is mainly produced by hepatocytes, but several studies indicate additional extrahepatic synthesis, e.g. in adipocytes or smooth muscle cells of the coronary arteries under inflammatory conditions^2,3^. Polymorphisms have been identified in the human *CRP* gene at locus 1q23.2 that are strongly associated with CRP values in human populations^4^. CRP as a member of the pentraxin family shows reactivity with ligands widely distributed in organisms, with inducing the complement system by interaction with the binding-sites^5^. Several inflammatory conditions such as rheumatoid arthritis, cardiovascular diseases, and infections show elevated expression of CRP^6^. Due to severe tissue damage like injury, infection or inflammation, CRP-concentration rises in a cytokine-mediated response within 24–72 h, while highest concentrations of CRP triggered by bacterial infections have been found in serum, increasing CRP values up to 1,000-fold^1,7,8^,. Plasma half-life of CRP is 18–20 h^9^.

CRP values in human populations are known to be influenced by age and sex, but also by factors such as lipid levels, blood pressure, body weight, estrogen (i.e., in hormone replacement therapy), nicotine- and alcohol consumption, which are in turn related to health behaviors, psychosocial stress and material living conditions and may potentially mediate the impact of socioeconomic factors on CRP^1,4^. Indicators of individual socioeconomic position (SEP), such as educational attainment and household income, are well-investigated determinants for diseases connected to inflammation and for inflammatory biomarkers including CRP^10–15^. For instance, Liu et al.^16^ have found that the influence of socioeconomic factors in childhood and adolescence plays a role for adult CRP values. In their study, an up to 25% elevated inflammatory level in the least advantaged group of participants (compared to the most advantaged) has been shown (24)^16^. In a meta-analysis, the association of educational degree and the complex regulatory inflammatory cascade trigger has been investigated with CRP showing the strongest social differences with higher CRP values in lower compared to higher educational groups, even after adjusting for health behaviors and body mass index^14^. Next to SEP playing an important role for the average values of CRP in blood serum of healthy subjects, variation in the *CRP* gene is a well-known determinant^17^. While in recent large scale genome-wide association studies (GWAS) up to 266 independent genetic loci have been reported to be associated with values of circulating CRP, genetic variants in the *CRP* gene locus continue to be among the strongest single genetic determinants of CRP values in humans^18,19^. It has been hypothesized that the incomplete penetrance of genetic variants of complex traits such as CRP values may at least partly be explained by gene by environment interactions^20^. For a range of complex traits that are associated with CRP values in human populations, gene by SEP interaction has been discussed^10,21–24^, with SEP serving as a context-defining indicator of social differences in non-genetic risk factors. While the association between low SEP and high CRP values has been observed in numerous studies and gene by SEP interactions have been shown for several inflammation-associated complex traits, the interaction between *CRP* locus genetic variation and SEP indicators has not been investigated to date.

The aim of the present study was to investigate to what extent the effect of allelic variation in the *CRP* gene single nucleotide polymorphism (SNP) rs4287174 on CRP values interacts with the SEP indicators education and income in a population-based study sample.

## Methods

### Study population

Baseline data of the Heinz Nixdorf Recall Study, a population-based prospective cohort, was used. The rationale and design of the study has been described in detail elsewhere^25^. In brief, 4,814 women and men between 45 and 74 years were randomly selected from mandatory registries of residence from the cities Bochum, Essen, and Mülheim/Ruhr within a large metropolitan region in the western part of Germany. The baseline response rate (recruitment period: December 2000 to June 2003) was 55.8% ^26^. From all participants written informed consent was obtained and the approval for the study was given by the ethics committee of the University of Duisburg-Essen in addition to extended quality management procedures and certification according to DIN ISO 9001:2000. The study adhered to the tenets of the declaration of Helsinki.

### Indicators of socioeconomic position (SEP)

Income categories and educational attainment assessed at the baseline examination in standardized face-to-face interviews were used as SEP indicators. Education (total years theoretically spent in education combining the highest formal school degree and highest vocational training) was categorized according to the International Standard Classification of Education^27^. For statistical analysis, four educational groups were created with the lowest educational group of ≤ 10 years of formal education equivalent to a minimum compulsory school attendance and no additional vocational degree and the highest education group of ≥ 18 years equivalent to a vocational training including additional qualification or a university degree. Income category was converted in the monthly household equivalent income calculated by dividing the total household net income of the participants by a weighting factor for each household member. Four groups were defined for income based on sex-specific quartiles. To investigate the individual contribution of SEP indicators education and income, they were analyzed separately^28,29^.

### Genetic data

SNP rs4287174 has been reported as lead variant in a recent 1000Genomes GWAS on chronic inflammation being in strong linkage disequilibrium (LD) with rs2794520 (r^2^ = 0.98), the lead variant at the *CRP* locus in other recent CRP GWAS^18,19^.Thus, rs4287174 was used as genetic marker for variation at the *CRP* locus in the study population with T coded as the CRP increasing allele (i.e., effect allele). The effect allele frequency in the analysis population was 0.65. Genotyping was performed using the Infinium Global Screening Array (GSA chip) by Illumina. No deviation from Hardy–Weinberg equilibrium was detected for rs4287174 genotypes.

### C-reactive protein

Blood serum high-sensitivity CRP (mg/dl) was measured using a standardized assay (Roche Diagnostics, Basel, Switzerland) at the central laboratory of the University Hospital of Essen, Germany^30^. Blood serum samples were analyzed within 12 h after being collected and stored at 4 °C. Lower limit of detection was 0.015 mg/dl. Participants below the detection limit were set to 0.010 mg/dl.

### Risk factors

Levels of total cholesterol were derived from blood serum samples by using standardized enzymatic methods and analyzed within 12 h after collection at the central laboratory of the University Hospital of Essen, Germany. Diabetes mellitus was defined as either of the following criteria: reported history of diabetes mellitus, taking glucose lowering drugs, having fasting blood glucose levels of > 125 mg/dL, or having nonfasting glucose levels of ≥ 200 mg/dL. Coronary artery calcification (CAC) was quantified at the baseline examination by noncontrast-enhanced electron beam computed tomography using a C-150 scanner (GE Imatron, South San Francisco, CA). A value of zero indicates nondetectable calcification. Current smoking was defined as smoking cigarettes during the last 12 months. Body mass index (BMI) was computed from standardized measurements of height and weight (kg/m^2^). Hypertension was defined as having a systolic value of ≥ 140 mm Hg or a diastolic value of ≥ 90 mm Hg or taking regular antihypertensive medication. Blood pressure was assessed using an automated oscillometric device (Omron HEM-705-CP) and calculated as the mean of the second and third values of 3 measurements. Physical activity was defined as no exercise versus exercise one and more times per week. Dietary intake was assessed by a validated 21-item food frequency questionnaire. Based on the food frequency questionnaire, a cardioprotective dietary pattern index was calculated to determine the quality of the participants’ diet (i.e., the higher the dietary pattern index score, the healthier the diet)^23^.

### Statistical analysis

**Fig. 1:**
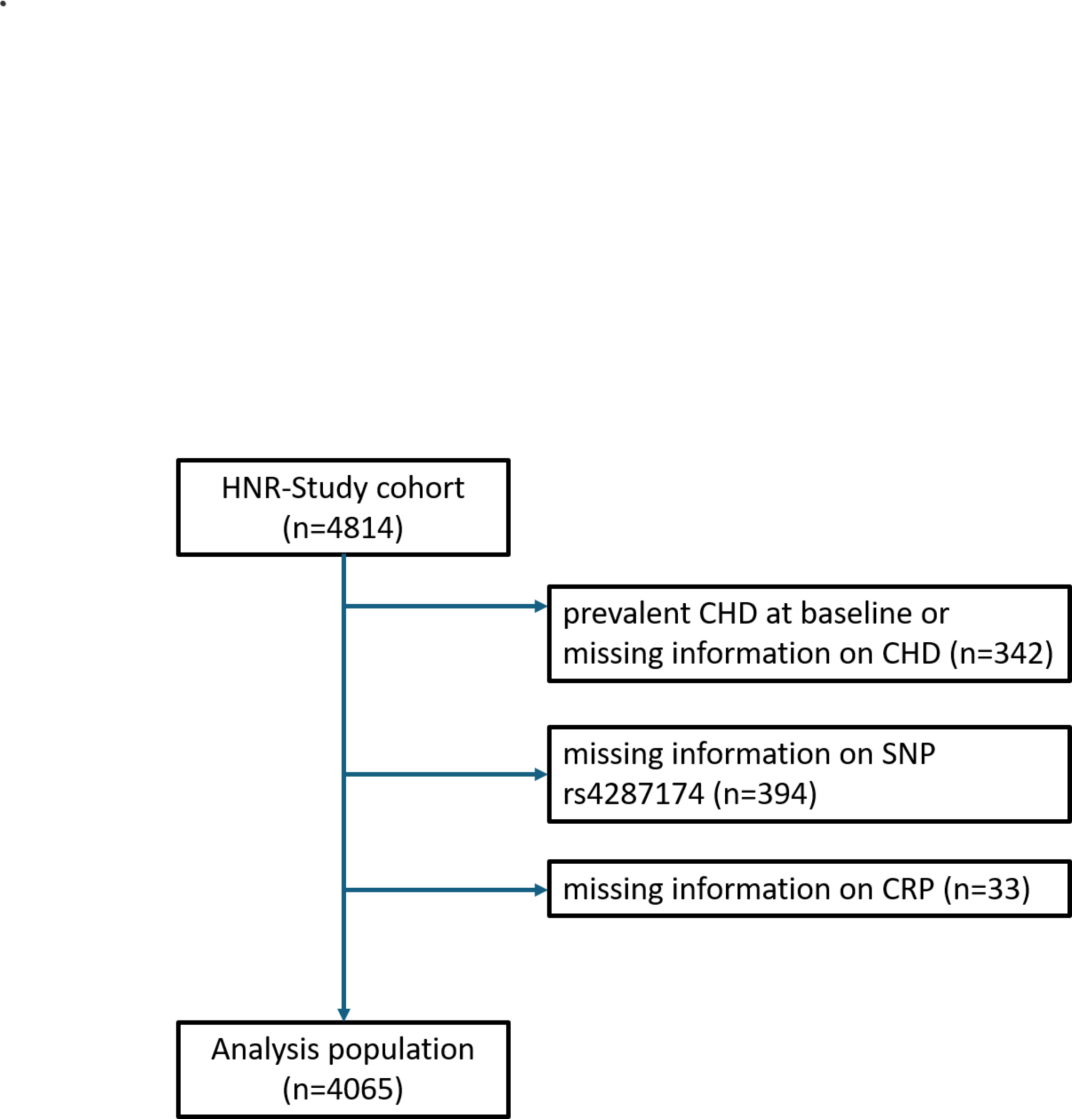
Participants of the Heinz Nixdorf Recall (HNR) study excluded from the analysis population (CHD: coronary heart disease; SNP: single nucleotide polymorphism; CRP: C-reactive Protein).

Out of the HNR-study cohort population (*n* = 4,814), participants with prevalent coronary heart diseases at baseline or missing information on prevalent coronary heart diseases (*n* = 342), as the main aim of the HNR study was the assessment of coronary artery calcification in a study sample free of coronary heart disease. Participants with missing data for SNP rs4287174 (*n* = 394) and for CRP values (*n* = 33) were also excluded from analysis (Fig. 1). Missing observations for education (*n* = 10), income (*n* = 263) and risk factors were excluded only from the respective analyses. Participants with prevalent coronary heart diseases at baseline or missing information on prevalent coronary heart diseases had moderately higher CRP values and income compared to the analysis population, while the distribution across educational groups did not differ (Suppl. Table 1). Participants with missing information on income were more likely to report low education and to be female, but did not differ in average CRP (Suppl. Table 2).

To characterize the analysis population (*n* = 4065), descriptive statistics were calculated for the whole analysis population as well as stratified by rs4287174 genotype. Age- and sex-adjusted linear regression models were fitted to quantify the associations of SEP indicators and SNP rs4287174 with CRP. A log_e_-transformation of (CRP + 1) was applied to approximate a symmetric distribution of the outcome. Effect size estimates and 95% confidence intervals (95%-CI) derived by linear regression models were presented back-transformed as exp[β] to interpret results on the original scale. Exp(beta) can be interpreted as the relative change in the geometric mean of CRP per unit increase in the independent variable. SNP rs4287174 was included in assuming a (log-)additive genetic model. To quantify the association of education and income with CRP, dummy variables were used with the highest SEP category as reference. To analyze the genetic effect of SNP rs4287174 on CRP in different SEP groups, age- and sex-adjusted linear regression models were fitted stratified by educational group and income quartile. The association between SEP indicators and CRP was also analyzed stratified by rs4287174 genotype. Age- and sex-adjusted linear regression models were fitted to assess strength of interaction between rs4287174 genotype and SEP indicators on CRP by including SEP group by rs4287174 interaction terms next to SEP and rs4287174 main effects in the model. Education and income were again analyzed separately and included as dummy variables with the highest category as reference. In addition, a single reference joint analysis was applied to assess the combined effect of SEP group and rs4287174 genotype on CRP. Dummy variables were created for all possible combinations of SEP group and rs4287174 genotype with the group of high SEP indicator and A allele CRP genotypes as reference. For this purpose, rs4287174 genotypes A/A and A/T were combined, as the group with high SEP and A/A genotype would have been too small to sufficiently serve as reference group with adequate statistical power.

SEP is likely to have no direct effect on CRP values, but its effect may be mediated by inflammatory risk factors associated with SEP. Potential gene by SEP interactions in the present study may thus be mediated by interactions of the *CRP* gene with risk factors affecting CRP values. Therefore, risk factors (i.e., body mass index (BMI), total cholesterol, diabetes mellitus, coronary artery calcification, current smoking, hypertension, diet, no exercise) were additionally included in the main interaction model as potential mediators (not confounders) of *CRP* gene by SEP interactions. According to Keller (2014)^31^,to properly adjust for covariates in a regression model including interaction terms it is necessary to not only include main effects but also covariate interaction terms. To avoid overfitting of regression models and to investigate, which one of the covariates may have a substantial impact on observed gene by SEP interactions, each covariate was included separately in the main interaction model. All analyses were performed using IBM ^®^ SPSS^®^ Statistics Version 27.

## Results

Sex and the average age did not strongly differ between the genotype groups (Table 1). The median CRP value showed an average of 0.15 (IQR: 0.07–0.32) mg/dl in the analysis population, while higher values were observed with each additional rs4287174 T allele. The distribution of household income (€/month) and years of education did not differ across genotype groups.

**Table 1:** Characteristics of the analysis population (*n* = 4065), stratified by SNP rs4287174 genotype.

Characteristic	All	A/A genotype	A/T genotype	T/T genotype
Number of subjects	4065	507	1838	1720
Women n (%)	2122 (52%)	272 (53%)	946 (51.4%)	904 (52.6%)
Age in years mean (± SD)	59.3 (± 7.8)	59.5 (± 7.8)	59.4 (± 7.8)	59.2 (± 7.65)
Education in years (n_miss_=10)				
≤ 10 n (%)	472 (11.6%)	60 (11.9%)	233 (12.7%)	179 (10.4%)
11–13 n (%)	2255 (55.6%)	292 (57.9%)	968 (52.8%)	995 (57.9%)
14–17 n (%)	894 (22.1%)	99 (19.7%)	427 (23.3%)	368 (21.4%)
≥ 18 n (%)	434 (10.7%)	53 (10.5%)	205 (11.2%)	176 (10.2%)
Income in €/month (n_miss_ = 263) median (Q1-Q3)	1449 (1108–1875)	1449 (1086–1875)	1449 (1107–1875)	1449 (1108–1875)
CRP in mg/dl median (Q1-Q3)	0.15 (0.07–0.32)	0.11 (0.05–0.24)	0.14 (0.07–0.29)	0.18 (0.09–0.40)
log_e_(CRP + 1) mean (± SD)	0.22 (± 0.25)	0.17 (± 0.19)	0.21 (± 0.24)	0.25 (± 0.28)
BMI (kg/m^2^) (n_miss_=20) Mean (± SD)	27.88 (± 4.6)	28.37 (± 4.83)	27.8 (± 4.62)	26.72 (± 4.58)
Diabetes mellitus n (%)	503 (12.4%)	53 (10.5%)	217 (11.8%)	233 (13.5%)
Dietary pattern index (n_miss_=74) Median (Q1-Q3)	13 (10–15)	13 (10–15)	13 (10–15)	12 (10–15)
Coronary artery calcification (n_miss_=868) Mean (± SD)	126.51 (± 357.37)	136.98 (± 366.24)	110,49 (± 292.34)	140,82 (± 413.91)
Total Cholesterol mg/dl (n_miss_=1) Median (Q1-Q3)	230 (205–255)	230 (205–255)	231 (204–255)	232 (206–255)
Current smoking n (%)	958 (23.6%)	120 (23.7%)	427 (23.2%)	411 (23.9%)
Hypertension n (%)	475 (11.7%)	48 (9.5%)	206 (11.2%)	221 (12.8%)
No exercise n (%)	1971 (48.5%)	241 (47.5%)	893 (48.6%)	837 (48.7%)

Legend: mean; standard deviation (SD), CRP (C-reactive Protein), log transformed CRP (logCRP), n _miss_:missing number in each group. T allele means the *CRP* risk effect allele and A the protective (reference) allele of rs4287174 genotype.

The main genetic effect of rs4287174 indicated a 1.045-fold (95%-CI: 1.033; 1.057) average CRP value per additional T allele (Table 2). The lowest education group showed on average 1.101-fold (95%-CI: 1.064; 1.139) CRP values, compared to the highest education group. Regarding income, 1.055-fold (95%-CI: 1.029; 1.081) average CRP values were observed in the lowest compared to the highest income quartile. For study participants in the intermediate SEP groups indication for higher average CRP values was also observed compared to the reference categories, leading to a negative trend in the magnitude of effect size estimates across SEP groups.

**Table 2:** Age- and sex-adjusted relative effect size estimates (exp(beta)) and 95% confidence intervals (95%-CI) for the association of SNP rs4287174 alleles, education groups (years) and income quartiles with CRP values assessed in separate linear regression models.

Characteristic	*n*	exp(ß)	95% CI	*p*
rs4287174*	4065	1.045	1.033; 1.057	3.763^− 14^
Education in years	4055			
≤ 10y		1.101	1.064; 1.139	4.156^− 08^
11-13y		1.064	1.036; 1.092	3.746^− 06^
14-17y		1.036	1.006; 1.066	0.017
≥ 18 y		reference		
Income in €/month	3802			
1st Quartile		1.055	1.029; 1.081	1.842^− 05^
2nd Quartile		1.034	1.010; 1.058	0.005
3rd Quartile		1.012	0.989; 1.035	0.305
4th Quartile		reference		

*Included in the analysis using an additive genetic model with T as the effect allele and A as the reference allele.

**Fig. 2:**
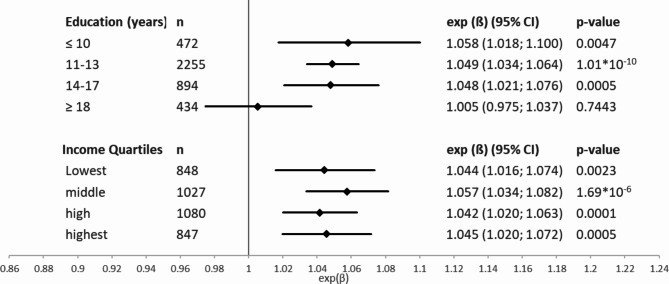
Age- and sex-adjusted relative genetic effects (exp(beta)) of SNP rs4287174 alleles on CRP values, stratified by education groups (years) and income quartiles in linear regression models; 95%-CI: 95% confidence interval.

The rs4287174 genetic effect on CRP stratified by education showed the strongest effect size estimate for the lowest education group with an exp(ß) of 1.058 (95%-CI: 1.018; 1.100), while in the highest education group the genetic effect nearly disappeared (exp(ß) of 1.005 (95%-CI: 0.975; 1.037)) (Fig. 2). A decrease in strength of effect size could be observed with increasing education, although in the intermediate education groups the effect size estimates were similar in magnitude (Fig. 2). When stratified by income quartiles, the strongest genetic effect could be observed in the second quartile with an exp(ß) of 1.057 (95%-CI: 1.034; 1.082), but no strong differences in the effect size estimates for the rs4287174 genetic effect on CRP were observed across income quartiles (Fig. 2).

When analyzing the association between education and CRP values stratified by rs4287174 genotype, the strongest effect size estimates were observed in the T/T genotype group showing an exp(ß) of 1.152 (95%-CI: 1.086;1.223) for the lowest compared with the highest education group (Suppl. Table 3).

The results in Table 3 indicated a positive interaction between rs4287174 alleles and low (≤ 10 years) as well as intermediate (11–13 years) education compared to the highest education group. The lower the educational level, the stronger were the effect size estimates for interaction by rs4287174 across educational groups. However, no interaction between effect alleles and income was found.

**Table 3:** Age- and sex- adjusted relative effect size estimates (exp(beta)) and corresponding 95% confidence intervals (95%-CI) for the interactions of rs4287174 alleles with education groups (years) and income quartiles on CRP values in separate linear regression models.

	exp(ß)	95% CI	*p*
**Education (n = 4055)**			
ß_0_	1.068	0.987 ; 1.157	0.104
Sex	0.985	0.969 ; 1.001	0.007
Age	1.002	1.001 ; 1.003	0.001
≤ 10y^¥^	1.028	0.958 ; 1.104	0.438
11-13y^¥^	1.005	0.951 ; 1.063	0.850
14-17y^¥^	0.981	0.921 ; 1.044	0.543
rs4287174^§^	1.005	0.970 ; 1.041	0.786
≤ 10y^¥^ * rs4287174^§^	1.056	1.005 ; 1.108	0.029
11-13y^¥^ * rs4287174^§^	1.044	1.005 ; 1.084	0.027
14-17y^¥^ * rs4287174^§^	1.043	0.999 ; 1.089	0.056
**Income (n = 3802)**			
ß_0_	0.998	0.926 ; 1.076	0.963
Sex	1.000	0.984 ; 1.016	0.978
Age	1.002	1.001 ; 1.003	0.001
1st Quartile^π^	1.055	1.001 ; 1.113	0.047
2nd Quartile^π^	1.020	0.970 ; 1.072	0.438
3rd Quartile^π^	1.016	0.967 ; 1.068	0.532
rs4287174^§^	1.044	1.018 ; 1.071	0.001
1st Quartile^π^ * rs4287174^§^	1.000	0.965 ; 1.036	0.992
2nd Quartile^π^ * rs4287174^§^	1.013	0.978 ; 1.048	0.477
3rd Quartile^π^* rs4287174^§^	0.998	0.965 ; 1.032	0.889

^§^ included in the analysis using an additive genetic model with T as the effect allele and A as the reference allele; ^¥^highest education group ≥ 18y used as reference; ^π^4th (highest) quartile was used as reference.

In the analysis of joint effects using a single reference group of participants within the highest education group and with A allele rs4287174 genotypes, effect size estimates showed a clear trend within and between education groups (Table 4). The lower the education group and the higher the number of CRP-increasing rs4287174 alleles the stronger the association with CRP. The strongest effect size estimate was observed for the group with the lowest education and two CRP-increasing rs4287174 alleles showing an exp(ß) of 1.156 (95%-CI: 1.102; 1.214) compared to the reference group.

**Table 4:** Age- and sex-adjusted single reference joint effects (exp(beta)) and corresponding 95% confidence intervals (95%-CI) for all possible combinations of education group and rs4287174 genotype on CRP values with the group of highest education and CRP genotypes A/A and A/T as reference.

Education and genotype	*n*	exp(ß)	95% CI	*p*
Education ≤ 10y				
T/T	179	1.156	1.102 ; 1.214	4.199^− 09^
A/A & A/T	293	1.073	1.028 ; 1.120	0.001
Education 11-13y				
T/T	995	1.105	1.067 ; 1.144	1.653^− 08^
A/A & A/T	1260	1.038	1.004 ; 1.074	0.029
Education 14-17y				
T/T	368	1.071	1.030 ; 1.115	0.001
A/A & A/T	526	1.017	0.979 ; 1.055	0.388
Education ≥ 18y				
T/T	176	1.007	0.960 ; 1.057	0.766
A/A & A/T	258	reference		

For the risk factors separately included in the main interaction model the effect size estimates for the rs4287174 by education interaction terms differed only slightly. For none of the risk factors an interaction with rs4287174 was indicated (Table 5).

**Table 5:** Age- and sex- adjusted relative effect size estimates (exp(beta)) and corresponding 95% confidence intervals (95%-CI) for the interactions of rs4287174 alleles with education groups (years) on CRP values additionally including risk factor main effects and rs4287174 by risk factor interactions terms in separate linear regression models.

	*n*	exp(ß)	(95% CI)	*p*
**Body mass index (BMI)**	4035			
**≤ 10y** ^**¥**^ *rs4287174 ^**§**^		1.065	1.015; 1.117	0.012
**11-13y** ^**¥**^ *rs4287174 ^**§**^		1.047	1.009; 1.087	0.015
**14-17y** ^**¥**^ *rs4287174 ^**§**^		1.046	1.004; 1.091	0.034
BMI*rs4287174		1.000	0.998; 1.002	0.993
**Total Cholesterol**	4054			
**≤ 10y** ^**¥**^ *rs4287174 ^**§**^		1.057	1.006; 1.108	0.028
**11-13y** ^**¥**^ *rs4287174 ^**§**^		1.044	1.005; 1.084	0.027
**14-17y** ^**¥**^ *rs4287174 ^**§**^		1.043	0.999; 1.089	0.056
Cholesterol*rs4287174		1.000	1.000; 1.000	0.911
**Diabetes mellitus**	4055			
**≤ 10y** ^**¥**^ *rs4287174 ^**§**^		1.053	1.004; 1.106	0.035
**11-13y** ^**¥**^ *rs4287174 ^**§**^		1.042	1.003; 1.082	0.034
**14-17y** ^**¥**^ *rs4287174 ^**§**^		1.040	0.997; 1.085	0.071
Diabetes*rs4287174		1.001	0.967; 1.037	0.949
**Coronary artery calcification (CAC)**	3193			
**≤ 10y** ^**¥**^ *rs4287174 ^**§**^		1.059	1.002; 1.119	0.041
**11-13y** ^**¥**^ *rs4287174 ^**§**^		1.031	0.990; 1.075	0.133
**14-17y** ^**¥**^ *rs4287174 ^**§**^		1.042	0.994; 1.091	0.085
CAC*rs4287174		1.000	1.000; 1.000	0.992
**Dietary pattern index**	3983			
**≤ 10y** ^**¥**^ *rs4287174 ^**§**^		1.053	1.002; 1,106	0.042
**11-13y** ^**¥**^ *rs4287174 ^**§**^		1.045	1.006; 1.085	0.024
**14-17y** ^**¥**^ *rs4287174 ^**§**^		1.044	1.000; 1.090	0.052
Diet*rs4287174		0.999	0.995; 1.003	0.587
**Current smoking**	4055			
**≤ 10y** ^**¥**^ *rs4287174 ^**§**^		1.055	1.005; 1.107	0.033
**11-13y** ^**¥**^ *rs4287174 ^**§**^		1.044	1.004; 1.084	0.029
**14-17y** ^**¥**^ *rs4287174 ^**§**^		1.042	0.998; 1.088	0.059
Active soking*rs4287174		1.006	0.980; 1.034	0.634
**Hypertension**	4055			
**≤ 10y** ^**¥**^ *rs4287174 ^**§**^		1.055	1.006; 1.108	0.029
**11-13y** ^**¥**^ *rs4287174 ^**§**^		1.044	1.005; 1.084	0.027
**14-17y** ^**¥**^ *rs4287174 ^**§**^		1.043	0.999; 1.089	0.056
RR*rs4287174		0.983	0.948; 1.019	0.355
**No exercise**	4055			
**≤ 10y** ^**¥**^ *rs4287174 ^**§**^		1.053	1.003; 1.106	0.037
**11-13y** ^**¥**^ *rs4287174 ^**§**^		1.042	1.003; 1.082	0.036
**14-17y** ^**¥**^ *rs4287174 ^**§**^		1.040	0.996; 1.085	0.072
Exercise *rs4287174		1.001	0.978; 1.024	0.952

^§^ included in the analysis using an additive genetic model with T as the effect allele and A as the reference allele; ^¥^highest education group ≥ 18y used as reference.

## Discussion

The results of the present study gave indication for interaction between allelic variation in the *CRP* gene SNP rs4287174 and education on CRP values in a population-based study sample. This was supported by (1) genetic effects stratified by education group showing stronger effect size estimates in groups of lower education, (2) positive rs4287174 T allele by low education interaction in linear regression models including interaction terms and (3) single reference joint effects showing the highest CRP values in the group with the lowest education and two rs4287174 T alleles. The observed interaction did not seem to be mediated by risk factors associated with SEP.

The results for the association of SEP indicators with CRP values are in line with previous studies that established the link between low SEP and higher CRP values in adult populations^14,32^. There are many hypotheses on the underlying cause of the association between SEP and negative health outcomes in general: lower education might for example correlate with limited knowledge about health behaviors and health and low health literacy, but also adverse housing and working conditions, reduced material resources and increased physical strain^11,28,29^. Low SEP is associated adverse lifestyle factors (e.g., poor diet, smoking, low physical activity) leading to a higher prevalence of adverse factors (e.g., hypertension, high total cholesterol, high BMI) that increase the risk for a range of common complex diseases. Such risk factors could act as mediators and account for the association of SEP with CRP values: Kershaw et al.^33^ assumed health behavior such as smoking, diet and physical activity would explain the major proportion of the association between SEP and CRP. According to a previous study low-grade inflammation (possibly indicated by elevated CRP values) might be the connection between well-known cardiovascular risk factors and cardiovascular disease^32^. In addition, according to the psychosocial explanation model for health inequalities also adverse psychosocial factors that are more prevalent in low SEP groups may directly increase CRP levels via psychoneuroendocrine and psychoneuroimmunological pathways^34^. In accordance with the present study results inflammation might also be a mediator between SEP and cardiovascular disease^32^.

The SNP rs4287174 showed a strong main effect on CRP values in this study, which is in accordance with the meta-analysis of Ligthart et al.^18^. However, the genotype is not solely responsible for basal CRP upregulation. Main results of the present study indicated that education may have an impact on the expression of the *CRP* genotype effect on CRP values. While stronger genetic effects were observed within groups of lower education, it is unlikely that education has a direct effect on the expression of genetic effects. Inflammatory risk factors more prevalent in lower SEP groups may potentially mediate the observed interaction. Several previous studies have identified factors associated with SEP interacting with the *CRP* genotype. Eiriksdottir et al.^35^ showed interaction between another SNP variant (rs1205) and BMI on circulating CRP values. The authors hypothesized that the adipose tissue might be producing a factor that modulates the expression of the *CRP* gene. Another study investigated the interaction of three *CRP* SNPs with plasma fatty acids on the inflammatory profile and presented positive results for rs1417938 ^36^. This was regarded as an indication for nutritional habits influencing the *CRP* gene expression, which is underlined by the results of Nienaber-Rousseau et al.^37^ showing interaction between several *CRP* SNPs and glucose fasting on inflammation. Yuan et al. (36)^38^ saw interaction of a CRP genetic risk score with copper, so occupational exposure might also play a role. All these factors – BMI, nutrition and occupational exposure – are known to be influenced by SEP^11^. They have also been discussed as potentially modifying epigenetic profiles, which could be hypothesized as one biological pathways for the observed interaction^39,40^. However, after including additional risk factors as potential mediators of the *CRP* gene by education interaction in the present analysis, the observed interaction did not seem to be mediated by these risk factors. As there was also no indication for interactions between the *CRP* gene and risk factors themselves, previous interactions reported for, e.g., BMI and diet, may have been confounded by SEP. In conclusion, there have to be other possible pathways through which SEP (as indicated by education in the present study) could affect the association of *CRP* genotype and circulating CRP levels.

In contrast to education, there was no indication for interaction between income and rs4287174 in this study. A possible explanation might be that the measurement of income is a snapshot which can alter due to changes in employment status (such as unemployment periods or retirement), while education is a constant characteristic usually completed early in life that reflects SEP in childhood, young adulthood and over early life course^28^. When Geyer et al.^29^ compared the SEP-indicators education, income and occupational position with regard to several health outcomes, differing results were observed related to the strength of associations. Education showed the strongest associations in the German study sample especially for diabetes mellitus and incident coronary events. They concluded that the investigated indicators reflect different dimensions of SEP with disparate effects on health outcomes. Income might represent dimensions of SEP which do not interact with *CRP* genotype, such as material resources and living conditions.

Besides the population-based study sample, the possibility to compare two different SEP indicators in the analyses is a strength of the present study. The sample size provided adequate statistical power for detecting the obtained genotype by education interaction effect size estimates, but was not sufficient for multiple testing of all CRP loci discovered to date. However, the evidence for interaction was not only based on estimating interaction terms but also on stratified and joint effect analyses. Results have to be replicated in additional study populations to check external validity. It would also be of interest, if interaction with SEP could be shown for other genetic loci associated with CRP. Additionally, due to the participants’ average age of about 60 years the impact of SEP on *CRP* gene expression in younger subjects remains unclear.

Despite these limitations, the current results gave supporting evidence for the interplay of SEP with *CRP* gene expression. We showed that a *CRP* genotype interacts with the SEP indicator education to influence individual CRP values in a population-based study sample and discussed possible pathways through which the influence might occur. While the effect of single common genetic variants on phenotypes such as CRP may be to small to be an intervention target or to be used for risk prediction in clinical routine, findings of this study highlight the strong impact of SEP even on genetically influenced aspects of health and underlines the importance of tackling health inequalities by reducing social inequalities.

## Electronic Supplementary Material

Below is the link to the electronic supplementary material.

Supplementary Material 1

## Data Availability

Due to data security reasons (i.e., data contain potentially participant identifying information), the Heinz Nixdorf Recall Study does not allow sharing data as a public use file. However, others can access the data used upon request, which is the same way the authors of the present paper obtained the data. Data requests can be addressed to the corresponding author at recall@uk-essen.de.

## References

[1] Sproston, N. R. & Ashworth, J. J. Role of C-reactive protein at sites of inflammation and infection. *Front. Immunol.* **9**10.3389/fimmu.2018.00754 (2018). 10.3389/fimmu.2018.00754 PMC5908901 29706967

[2] Calabró, P., Willerson, J. T. & Yeh, E. T. H. Inflammatory cytokines stimulated C-reactive protein production by human coronary artery smooth muscle cells. *Circulation* **108** (16), 1930–1932. 10.1161/01.CIR.0000096055.62724.C5 (2003). 14530191 10.1161/01.CIR.0000096055.62724.C5

[3] Calabro, P., Chang, D. W., Willerson, J. T. & Yeh, E. T. H. Release of C-reactive protein in response to inflammatory cytokines by human adipocytes: linking obesity to vascular inflammation. *J. Am. Coll. Cardiol.* **46** (6), 1112–1113. 10.1016/j.jacc.2005.06.017 (2005). 16168299 10.1016/j.jacc.2005.06.017

[4] Hage, F. G. & Szalai, A. J. C-reactive protein gene polymorphisms, C-reactive protein blood levels, and cardiovascular disease risk. *J. Am. Coll. Cardiol.* **50** (12), 1115–1122. 10.1016/j.jacc.2007.06.012 (2007). 17868801 10.1016/j.jacc.2007.06.012

[5] Black, S., Kushner, I. & Samols, D. C-reactive protein. *J. Biol. Chem.* **279**(47), 48487–48490. 10.1074/jbc.R400025200 (2004). 10.1074/jbc.R400025200 15337754

[6] Du Clos, T. W. & Mold, C. C-reactive protein: an activator of innate immunity and a modulator of adaptive immunity. *Immunol. Res.* **30** (3), 261–277. 10.1385/IR:30:3:261 (2004). 15531769 10.1385/IR:30:3:261

[7] Thompson, D., Pepys, M. B. & Wood, S. P. The physiological structure of human C-reactive protein and its complex with phosphocholine. *Structure* **7** (2), 169–177. 10.1016/S0969-2126(99)80023-9 (1999). 10368284 10.1016/S0969-2126(99)80023-9

[8] Ciubotaru, I., Potempa, L. A. & Wander, R. C. Production of modified C-reactive protein in U937-derived macrophages. *Exp. Biol. Med. (Maywood)*. **230** (10), 762–770. 10.1177/153537020523001010 (2005). 16246904 10.1177/153537020523001010

[9] Ridker, P. M. Clinical application of C-reactive protein for cardiovascular disease detection and prevention. *Circulation* **107** (3), 363–369. 10.1161/01.cir.0000053730.47739.3c (2003). 12551853 10.1161/01.cir.0000053730.47739.3c

[10] Nagar, S. D. et al. Comparing genetic and socioenvironmental contributions to ethnic differences in C-reactive protein. *Front. Genet.* **12**, 738485. 10.3389/fgene.2021.738485 (2021). 34733313 10.3389/fgene.2021.738485 PMC8558394

[11] Pampel, F. C., Krueger, P. M. & Denney, J. T. Socioeconomic disparities in health behaviors. *Annu. Rev. Sociol.* **36**, 349–370. 10.1146/annurev.soc.012809.102529 (2010). 21909182 10.1146/annurev.soc.012809.102529 PMC3169799

[12] Johnson, W. & Krueger, R. F. Genetic effects on physical health: Lower at higher income levels. *Behav. Genet.* **35** (5), 579–590. 10.1007/s10519-005-3598-0 (2005). 16184486 10.1007/s10519-005-3598-0

[13] Johnson, W. et al. Education reduces the effects of genetic susceptibilities to poor physical health. *Int. J. Epidemiol.* **39** (2), 406–414. 10.1093/ije/dyp314 (2010). 19861402 10.1093/ije/dyp314

[14] Maurel, M. et al. Patterning of educational attainment across inflammatory markers: Findings from a multi-cohort study. *Brain Behav. Immun.* **90**, 303–310. 10.1016/j.bbi.2020.09.002 (2020). 32919037 10.1016/j.bbi.2020.09.002 PMC8140486

[15] Johnson, W., Kyvik, K. O., Skytthe, A., Deary, I. J. & Sørensen, T. I. A. Education modifies genetic and environmental influences on BMI. *PLoS One*. **6** (1), e16290. 10.1371/journal.pone.0016290 (2011). 21283825 10.1371/journal.pone.0016290 PMC3023797

[16] Liu, R. S. et al. Socioeconomic status in childhood and C reactive protein in adulthood: A systematic review and meta-analysis. *J. Epidemiol. Commun. Health*. **71** (8), 817–826. 10.1136/jech-2016-208646 (2017). 10.1136/jech-2016-208646 PMC5843476 28490476

[17] Kushner, I., Rzewnicki, D. & Samols, D. What does minor elevation of C-reactive protein signify? *Am. J. Med.* **119** (2), 166e17–166e28. 10.1016/j.amjmed.2005.06.057 (2006). 10.1016/j.amjmed.2005.06.057 16443421

[18] Ligthart, S. et al. Genome analyses of 200,000 individuals identify 58 loci for chronic inflammation and highlight pathways that link inflammation and complex disorders. *Am. J. Hum. Genet.* **103** (5), 691–706. 10.1016/j.ajhg.2018.09.009 (2018). 30388399 10.1016/j.ajhg.2018.09.009 PMC6218410

[19] Said, S. et al. Genetic analysis of over half a million people characterises C-reactive protein loci. *Nat. Commun.* **13** (1), 2198. 10.1038/s41467-022-29650-5 (2022). 35459240 10.1038/s41467-022-29650-5 PMC9033829

[20] Manolio, T. A. et al. Finding the missing heritability of complex diseases. *Nature* **461** (7265), 747–753. 10.1038/nature08494 (2009). 19812666 10.1038/nature08494 PMC2831613

[21] Buchwald, S. et al. Tooth loss and periodontitis by socio-economic status and inflammation in a longitudinal population-based study. *J. Clin. Periodontol*. **40** (3), 203–211. 10.1111/jcpe.12056 (2013). 23379538 10.1111/jcpe.12056

[22] Myburgh, P. H., Nienaber-Rousseau, C., Kruger, I. M. & Towers, G. W. Education, smoking and CRP genetics in relation to C-reactive protein concentrations in Black South Africans. *IJERPH* **17** (18), 6646. 10.3390/ijerph17186646 (2020). 32933066 10.3390/ijerph17186646 PMC7558133

[23] Schmidt, B. et al. Socioeconomic status interacts with the genetic effect of a chromosome 9p21.3 common variant to influence coronary artery calcification and incident coronary events in the Heinz Nixdorf Recall Study (risk factors, evaluation of coronary calcium, and lifestyle). *Circ. Cardiovasc. Genet.* **10** (2). 10.1161/CIRCGENETICS.116.001441 (2017). 10.1161/CIRCGENETICS.116.001441 28411192

[24] Frank, M. et al. A genetic sum score of risk alleles associated with body mass index interacts with socioeconomic position in the Heinz Nixdorf Recall Study. *PLoS One*. **14** (8), e0221252. 10.1371/journal.pone.0221252 (2019). 31442235 10.1371/journal.pone.0221252 PMC6707579

[25] Schmermund, A. et al. Assessment of clinically silent atherosclerotic disease and established and novel risk factors for predicting myocardial infarction and cardiac death in healthy middle-aged subjects: Rationale and design of the Heinz Nixdorf RECALL Study. Risk factors, evaluation of coronary calcium and lifestyle. *Am. Heart J.* **144** (2), 212–218. 10.1067/mhj.2002.123579 (2002). 12177636 10.1067/mhj.2002.123579

[26] Stang, A. et al. Baseline recruitment and analyses of nonresponse of the Heinz Nixdorf recall study: Identifiability of phone numbers as the major determinant of response. *Eur. J. Epidemiol.* **20** (6), 489–496. 10.1007/s10654-005-5529-z (2005). 16121757 10.1007/s10654-005-5529-z

[27] *International Standard Classification of Education (ISCED) 2011*. (UNESCO, 2012).

[28] Galobardes, B., Shaw, M., Lawlor, D. A., Lynch, J. W. & Davey Smith, G. Indicators of socioeconomic position (part 1). *J. Epidemiol. Commun. Health*. **60** (1), 7–12. 10.1136/jech.2004.023531 (2006). 10.1136/jech.2004.023531 PMC2465546 16361448

[29] Geyer, S., Hemström, O., Peter, R. & Vågerö, D. Education, income, and occupational class cannot be used interchangeably in social epidemiology. Empirical evidence against a common practice. *J. Epidemiol. Commun. Health*. **60** (9), 804–810. 10.1136/jech.2005.041319 (2006). 10.1136/jech.2005.041319 PMC2566032 16905727

[30] Möhlenkamp, S. et al. Quantification of coronary atherosclerosis and inflammation to predict coronary events and all-cause mortality. *J. Am. Coll. Cardiol.* **57** (13), 1455–1464. 10.1016/j.jacc.2010.10.043 (2011). 21435514 10.1016/j.jacc.2010.10.043

[31] Keller, M. C. Gene × environment interaction studies have not properly controlled for potential confounders: The problem and the (simple) solution. *Biol. Psychiatry*. **75** (1), 18–24. 10.1016/j.biopsych.2013.09.006 (2014). 24135711 10.1016/j.biopsych.2013.09.006 PMC3859520

[32] Nazmi, A. & Victora, C. G. Socioeconomic and racial/ethnic differentials of C-reactive protein levels: A systematic review of population-based studies. *BMC Public. Health*. **7**, 212. 10.1186/1471-2458-7-212 (2007). 17705867 10.1186/1471-2458-7-212 PMC2018719

[33] Kershaw, K. N., Mezuk, B., Abdou, C. M., Rafferty, J. A. & Jackson, J. S. Socioeconomic position, health behaviors, and C-reactive protein: A moderated-mediation analysis. *Health Psychol.* **29** (3), 307–316. 10.1037/a0019286 (2010). 20496985 10.1037/a0019286 PMC2881158

[34] Chandola, T., Heraclides, A. & Kumari, M. Psychophysiological biomarkers of workplace stressors. *Neurosci. Biobehav Rev.* **35** (1), 51–57. 10.1016/j.neubiorev.2009.11.005 (2010). 19914288 10.1016/j.neubiorev.2009.11.005 PMC2891393

[35] Eiriksdottir, G. et al. The interaction of adiposity with the CRP gene affects CRP levels: Age, gene/environment susceptibilty-Reykjavik Study. *Int. J. Obes.* **33** (2), 267–272. 10.1038/ijo.2008.274 (2009). 10.1038/ijo.2008.274 PMC3149889 19139754

[36] Oki, E. et al. Interaction of SNP in the CRP gene and plasma fatty acid profile in inflammatory pattern: A cross-sectional population-based study. *Nutrition* **32** (1), 88–94. 10.1016/j.nut.2015.07.015 (2016). 26456189 10.1016/j.nut.2015.07.015

[37] Nienaber-Rousseau, C. et al. Interactions between C-reactive protein genotypes with markers of nutritional status in relation to inflammation. *Nutrients* **6** (11), 5034–5050. 10.3390/nu6115034 (2014). 25393688 10.3390/nu6115034 PMC4245578

[38] Yuan, Y. et al. Multiple plasma metals, genetic risk and serum C-reactive protein: A metal-metal and gene-metal interaction study. *Redox Biol.* **29**, 101404. 10.1016/j.redox.2019.101404 (2020). 31926627 10.1016/j.redox.2019.101404 PMC6921203

[39] Kim, K-N., Lee, M-R., Lim, Y-H. & Hong, Y-C. Blood lead levels, iron metabolism gene polymorphisms and homocysteine: A gene-environment interaction study. *Occup. Environ. Med.* **74** (12), 899–904. 10.1136/oemed-2017-104375 (2017). 28775131 10.1136/oemed-2017-104375

[40] Uher, R. Gene-environment interactions in common mental disorders: An update and strategy for a genome-wide search. *Soc. Psychiatry Psychiatr .Epidemiol.* **49** (1), 3–14. 10.1007/s00127-013-0801-0 (2014). 24323294 10.1007/s00127-013-0801-0

