# Supplementary material for: Socioeconomic position interacts with the genetic effect of a CRP gene common variant to influence C-reactive protein values
Source: Sci Rep. 2024 Dec 23;14:30612. doi: 10.1038/s41598-024-83437-w (PMC11666539; doi:10.1038/s41598-024-83437-w)
Supplement: Supplementary file 1 — Supplementary Material 1 [file 41598_2024_83437_MOESM1_ESM.docx]

**Supplemental Material**

**Suppl. Table 1: Characteristics of study participants with prevalent coronary heart diseases at baseline or missing information on prevalent coronary heart diseases (n=342).**

| Number of subjects | 342 |
| --- | --- |
| Women n (%) | 75 (21.9%) |
| Age in years mean (±SD) | 64.17 (±7.16) |
| Education in years (n_miss_=5) |  |
| ≤10 n (%) | 39 (11.4%) |
| 11-13 n (%) | 179 (52.3%) |
| 14-17 n (%) | 87 (25.4%) |
| ≥18 n (%) | 32 (9.4%) |
| Income in €/month (n_miss_=17) median (Q1-Q3) | 1313 (937-1874 |
| CRP in mg/dl median (Q1-Q3) (n_miss_=4) | 0.20 (0.09-0.44) |
| logCRP, M (± SD) (n_miss_=4) | 0.28 (±0.31) |

Legend: mean (M); standard deviation (SD), CRP (C-reactive Protein), n_miss_: missing number in each group_._

**Suppl. Table 2: Characteristics of study participants with missing information on household income (n=310).**

| Number of subjects | 310 |
| --- | --- |
| Women n (%) | 209 (67%) |
| Age in years, mean (±SD) | 61 (±7.4) |
| Education in years (n_miss_=15) |  |
| ≤10 y n (%) | 38 (12.26%) |
| 11-13 y n (%) | 192 (61.9%) |
| 14-17 y n (%) | 38(12.26%) |
| ≥18 y n (%) | 27 (8.71%) |
| CRP in mg/dl median (Q1-Q3) (n_miss_=5) | 0.16 (0.07-0.35) |
| logCRP, M (± SD) | 0.22 (±0.22) |

Legend: mean (M); standard deviation (SD), CRP (C-reactive Protein), n _miss_ : missing number in each group_._

**Suppl. Table 3: Age- and sex-adjusted relative effect sizes estimates (exp(ß)) and 95% confidence intervals (95%-CI) for the association of education groups (years; highest education group ≥18y used as reference) with CRP values stratified by rs4287174 genotype in linear regression models.**

| **Education by genotype** | **n** | **exp(ß)** | **95% CI** | ***p*** |
| --- | --- | --- | --- | --- |
| rs4287174 A/A | 504 |  |  |  |
| ≤10y |  | 1.033 | 0.960; 1.112 | 0.387 |
| 11-13y |  | 1.008 | 0.953; 1.067 | 0.771 |
| 14-17y |  | 0.972 | 0.912; 1.035 | 0.371 |
| rs4287174 A/T | 1833 |  |  |  |
| ≤10y |  | 1.079 | 1.029; 1.131 | 0.002 |
| 11-13y |  | 1.046 | 1.008; 1.084 | 0.016 |
| 14-17y |  | 1.028 | 0.988; 1.070 | 0.172 |
| rs4287174 T/T | 1718 |  |  |  |
| ≤10y |  | 1.152 | 1.086; 1.223 | 3.350^-06^ |
| 11-13y |  | 1.099 | 1.051; 1.150 | 4.204^-05^ |
| 14-17y |  | 1.063 | 1.011; 1.117 | 0.016 |

*highest education group ≥18y used as reference
